# Innate Immune Sensing of Parapoxvirus Orf Virus and Viral Immune Evasion

**DOI:** 10.3390/v17040587

**Published:** 2025-04-19

**Authors:** Basheer A. AlDaif, Stephen B. Fleming

**Affiliations:** Virus Research Unit, Department of Microbiology and Immunology, University of Otago, Dunedin 9016, New Zealand; basheer.al-daif@postgrad.otago.ac.nz

**Keywords:** Poxvirus, parapoxvirus, orf virus, innate immunity, innate sensing, interferon, immune evasion

## Abstract

Orf virus (ORFV) is the type species of *Parapoxvirus* of the *Poxviridae* family that induces cutaneous pustular skin lesions in sheep and goats, and causes zoonotic infections in humans. Pattern recognition receptors (PRRs) sense pathogen-associated molecular patterns (PAMPs), leading to the triggering of the innate immune response through multiple signalling pathways involving type I interferons (IFNs). The major PAMPs generated during viral infection are nucleic acids, which are the most important molecules that are recognized by the host. The induction of type l IFNs leads to activation of the Janus kinase (JAK)-signal transducer activator of transcription (STAT) pathway, which results in the induction of hundreds of interferon-stimulated genes (ISGs), many of which encode proteins that have antiviral roles in eliminating virus infection and create an antiviral state. Genetic and functional analyses have revealed that ORFV, as found for other poxviruses, has evolved multiple immunomodulatory genes and strategies that manipulate the innate immune sensing response.

## 1. Introduction

The *Parapoxvirus* genus (family *Poxviridae*, subfamily *Chordopoxvirus*) includes Orf virus (ORFV), Pseudocowpoxvirus (PCPV), Bovine popular stomatitis virus (BPSV) and Parapoxvirus of red deer in New Zealand (PVNZ). ORFV is the type species of the *Parapoxvirus* genus and the causative agent of Orf disease, a contagious debilitating skin condition that induces pustular skin lesions in sheep and goats and causes zoonotic infection in humans [1,2]. The symptoms of Orf disease in humans start as erythema, vesicles, pustules, and finally scabs. The lesions typically resolve within 6 to 12 weeks. In addition, ORFV is known to cause severe disease in those that are immunocompromised [1,2]. In these cases, the virus causes large vascularized tumour-like lesions of the skin [3,4].

Poxviruses have large linear dsDNA genomes and the basic genetic structure of a poxvirus was first shown for vaccinia virus (VACV), which has a genome of approximately 190 kbp [5]. VACV is a member of the Orthopoxvirus genus, the prototype of the poxvirus family, and the most extensively studied of the poxviruses. Poxviruses encode almost all factors required for replication [6,7]. All other poxviruses are essentially similar, but the genetic differences mainly lie within the termini of the genome. The genomes of several ORFV strains have been fully sequenced [8,9]. It was predicted that the ORFV genome contains 132 genes and the distribution of these genes is typical of poxviruses, with the central region containing genes essential for its life cycle, and the terminal regions containing host range restriction or virulence genes that are non-essential for growth in cell culture [1,2,10,11].

ORFV infects skin-resident keratinocytes and replicates exclusively in the cytoplasm, causing a localized infection that persists for weeks [12,13,14]. Keratinocytes reside in the epidermal layer of skin, constituting 90% of epidermal cells. They act as immune sentinels and have the capability to activate the innate immune response [15,16,17,18,19,20]. Innate immunity is a first line of defence and an immediate non-specific response against viral infection. Although an immune response is generated against ORFV infection, the virus has the ability to repeatedly infect its host [21,22]. This strongly indicates that ORFV expresses factors that antagonize this response. In this review, our current knowledge of strategies employed by ORFV to antagonize the innate immune sensing response will be reviewed.

## 2. Innate Immune Sensing

Host cells adopt multiple defence mechanisms to respond to invading viruses. Innate immunity is a first line of defence and an immediate response against viral infection [23]. A key step in this response is the detection of virus infection. Sensing pathogens by innate immunity is mediated by host pattern recognition receptors (PRRs) that recognize pathogen-associated molecular patterns (PAMPs). The major PAMPs generated during viral infection are nucleic acids and are the most important molecules that are recognized by the host [24].

**Figure 1:**
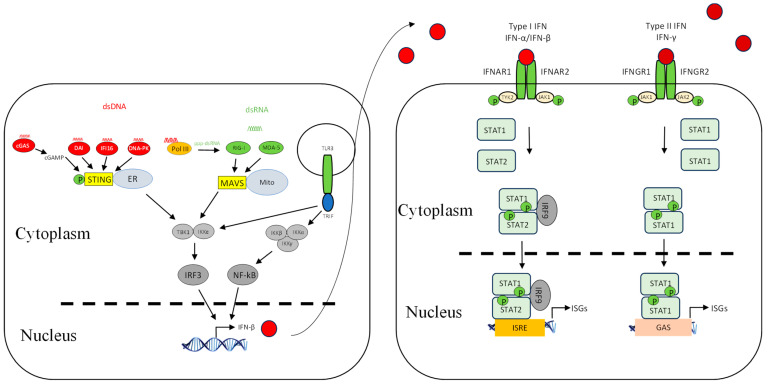
Interferon induction and signalling pathways. Shown are DNA- and RNA-sensing mechanisms that lead to IFN signalling. The DNA sensors cGAS, DAI, IFI16, DNA-PK, and Pol III are localized in the cytosol and utilize the adaptor STING residing on the endoplasmic reticulum. They detect DNA in the cytosol and trigger STING-dependent signalling, leading to the activation of TBK1/IKKε and IRF3 that results in IFN-β expression. cGAS synthesizes the production of cGAMP dinucleotides that bind STING. RIG-like receptors (RLRs) are localized in the cytosol and utilize the adaptor MAVS. The RIG-I receptor senses short dsRNA and also ppp-dsRNA derived from polymerase III-transcribed poly (dA:dT), whereas MDA5 senses long dsRNA. Upon ligand binding, RLRs engage the adaptor MAVS located on the outer membrane of mitochondria, which leads to downstream signalling via TBK1/IKKε and thus phosphorylation of IRF3 followed by IFN expression. TLR3 is localized in the endosome and utilizes the adaptor TRIF. Upon recognizing dsRNA, it can lead to IFN-β expression through TBK1/IKKβ-IRF3 and the IKKα/IKKβ/IKKγ-NF-κB axis. Upon IFN induction, a second line of signalling is initiated in which the induced IFNs interact with IFN receptors, in an autocrine or paracrine manner, leading to transcription of a diverse set of genes called IFN-stimulated genes via the Janus kinase (JAK)-signal transducer activator of transcription (STAT) pathway. Red circle is type I IFN, darker red circle is type II IFN, dashed line is nuclear membrane.

There are many receptors responsible for triggering the innate immune response and can be classified based on their subcellular localization and the distinct virus-derived molecules they recognize. C-type lectin receptors (CLRs) and Toll-like receptors (TLRs) are endosomal or membrane-bound receptors, whereas nucleotide-binding oligomerization domain (NOD)-like receptors (NLRs), AIM2-like receptors (ALRs), and retinoic acid inducible gene (RIG-I)-like receptors (RLRs) are cytosolic (Figure 1). Each class of these receptors activates specific signalling cascades, which, in turn, activate specific transcription factors to induce the expression of target genes, namely, type I IFNs and proinflammatory cytokines.

Among those receptors, the RIG-I-like receptor family is the main cytosolic RNA sensor. It consists of three groups of molecules: RIG-I, melanoma differentiation associated gene 5 (MDA5), and laboratory of genetic and physiology 2 (LGP2) [25]. In addition, there are other molecules that act as sensors for foreign dsRNAs: protein kinase R (PKR) and 2′-5′-Oligoadenylate Synthetase (OAS)/RNaseL. They are present in most cell types at basal levels and involve the inhibition of protein synthesis and RNA degradation, respectively [26,27,28].

In addition, host cells employ a number of DNA sensors that can detect DNA in the cytosol (Figure 1). These include DNA-dependent activator of interferon-regulatory factor (DAI) [29], RNA Polymerase III/RIG-I (RNA Pol III) [30,31,32,33], PYHIN family protein (IFI16/p204) [34,35], DExD/H-Box Helicases (DDX) protein [36], DNA-dependent protein kinase (DNA-PK) [37], Cyclic GMP-AMP (2′3′-cGAMP) [38], and Cyclic GMP-AMP Synthase (cGAS) [39,40,41].

Once the sensors are activated, RIG-I and MDA5 interact with mitochondrial antiviral signalling protein (MAVS) [42,43,44], whereas cytosolic DNA sensors interact with stimulator of interferon genes (STINGs) [45,46] (Figure 1). The cytosolic PRR-mediated signalling pathways converge at the IKK-related serine/threonine kinases TBK1/IKKε, which then phosphorylate and activate IRF3 and IRF7 [47,48,49] (Figure 1). TLR9 recruits MyD88 to transmit cellular signalling, leading to the activation of IRF7 and NF-κB [24,50], whereas TLR3 recruits TRIF to mediate signalling, leading to IRF3 and NF-κB activation [51,52,53]. The complex of TBK1/IKKε and associated subunits is important for the activation of IRF3 and IRF7 [54], whereas the complex of IKKα/IKKβ/IKKγ is important for the activation of NF-κB. Upon IRF3/IRF7 and NF-κB activation, they translocate to the nucleus and bind to their respective regulatory elements, culminating in the induction of type I IFNs and inflammatory cytokines, respectively [55]. The activated factors translocate to the nucleus, where they bind to the promoter regions of IFNs and proinflammatory cytokines for activation.

Upon IFN induction, a second line of signalling is initiated in which the induced IFNs interact with IFN receptors (IFNRs), in an autocrine or paracrine manner, leading to the transcription of a diverse set of genes called IFN-stimulated genes (ISGs) via the Janus kinase (JAK)-signal transducer activator of transcription (STAT) pathway (Figure 1). These ISGs are involved in eliminating viral infection from infected cells and conferring resistance to neighbouring cells. The established antiviral state will inhibit viral replication at various stages [27,56]. Some ISGs are induced directly by viral infection, but less effectively than the IFN response itself; however, their induction is amplified significantly by IFNs [57,58,59,60].

Among all of the TLRs characterized to date, only TLR-3, -7, and -8 are known to detect viral RNA [50,61,62]. TLR-7 and TLR-8 recruit Myeloid differentiation primary-response gene 88 (MyD88) to transmit cellular signalling, leading to the activation of transcription factors including IFN regulatory factors (IRFs), nuclear factor kappa B (NF-κB), and activating transcription factor 2 (ATF2)/c-Jun [63], whereas TLR3 recruits TIR-domain-containing adapter-inducing interferon-β (TRIF) to mediate signalling, leading to the activation of two transcription factors, IRF3 and NF-κB, resulting in the induction of type I IFNs and proinflammatory cytokines [51,64,65]. TLR7 is mostly found in plasmacytoid dendritic cells, and TLR8 is mainly expressed in myeloid dendritic cells and monocytes. However, TLR3 is ubiquitously expressed and detects viral dsRNA generated during virus replication and its synthetic analogue Poly (I:C) [66]. TLRs are not important in other cell types for the production of type I IFNs in response to viral infection; instead, cytosolic RNA sensors are essential [67].

## 3. Detection of Parapoxviruses by Innate Immune Sensing Receptors

Poxviruses replicate in the cytoplasm of infected cells, making their nucleic acids targets of cytosolic PRRs. After the virion core is released into the cytoplasm and before complete uncoating, early transcribed mRNAs are extruded from the core into the cytoplasm to be translated [7,68]. Early gene expression then ceases when the core is completely uncoated, and the viral genome is released into the cytoplasm, in which intermediate gene transcription and replication of the genome is initiated. Both the exposed viral RNA and DNA become targets of cellular receptors. Little is known about the specific detection of parapoxviruses by innate immune sensing at this time; however, based on the findings for other poxviruses in particular VACV and DNA viruses, it is more than likely that the same cytosolic RNA receptors and DNA receptors will have a role.

### 3.1. Intracellular Detection of Viral RNA

During replication, poxviruses produce appreciable amounts of dsRNA in the cytoplasm as a result of convergent transcription [69,70,71,72]. In addition, the generation of dsRNA can be produced from dsDNA by cellular RNA polymerase III (Pol III) [30,32]. The role of Pol III on DNA virus sensing was shown to mediate an antiviral response against VACV [32]. Not surprisingly, dsRNA generated from poxvirus replication can be a target for cytosolic RNA sensors. VACV dsRNA was shown to be detected by RIG-I and MDA5, thus activating an antiviral response [73,74]. Furthermore, MYXV elicited an IFN response via RIG-I-dependent sensing [75]. Protein kinase receptor (PKR) is an intracellular sensor of stress manifested during viral infection. Virally produced dsRNA activates PKR, which arrests protein synthesis by phosphorylating the alpha subunit of the translation initiation factor elF2. In addition, PKR is a key component of the IFN antiviral response against poxviruses. It was shown that PKR induced MDA5-mediated IFN-β induction during VACVΔE3L infection [76]. Interestingly, a homolog of VACV-E3L is found in ORFV, ORF020, that has been shown to bind dsRNA and inhibit the activation of PKR [77,78]. In addition, OAS and RNase L are dsRNA sensors that have antiviral roles [79] that have been illustrated by the finding that RNase L knock-out mice were susceptible to VACV infection [80,81]. It is likely that the range of RNA sensors that detect orthopoxviruses will be important for parapoxvirus detection.

### 3.2. Intracellular Detection of Viral DNA

Several studies have shown that viral genomic DNA can elicit an IFN response, and cytosolic PRRs are more important in detecting DNA viruses in the cytoplasm [82,83,84]. Although a number of cytosolic DNA sensors have been shown to have a role in a DNA-dependent IFN response, only a few have been implicated in poxvirus recognition.

Of all the DNA receptors identified at this time, cGAS appears to be critical for poxvirus detection. cGAS is an essential STING-dependent cytosolic DNA receptor, as proven by the induction of type I IFNs being severely impaired in several cell types (fibroblasts, macrophages, and DCs) lacking cGAS [40]. cGAS is required to detect DNA viruses such as HSV-1, KSHV, ECTV, and VACV [40,41,46,85,86]. It was shown to be the most potent inducer of IFN-β expression in comparison with other cytosolic DNA receptors such as DAI, IFI16, and DDX41 [39,40,41]. As eluded to above, RNA Pol III is also considered to be a DNA sensor for some DNA viruses including HSV-1, EBV, and VACV [32,33]. IFI16 is a DNA sensor that shuttles from the nucleus to viral factories for viral DNA detection. HSV-1 and VACV infection, but not their transfected DNA, can activate IFI16 that leads to IFN-β induction via IRF3 [35,87]. DNA-PK, besides its DNA repair activity, can sense cytosolic DNA and initiate an immune response to VACV [37].

## 4. Innate Immune Evasion Strategies by ORFV

### 4.1. Evasion of RNA-Dependent Sensing

**Figure 2:**
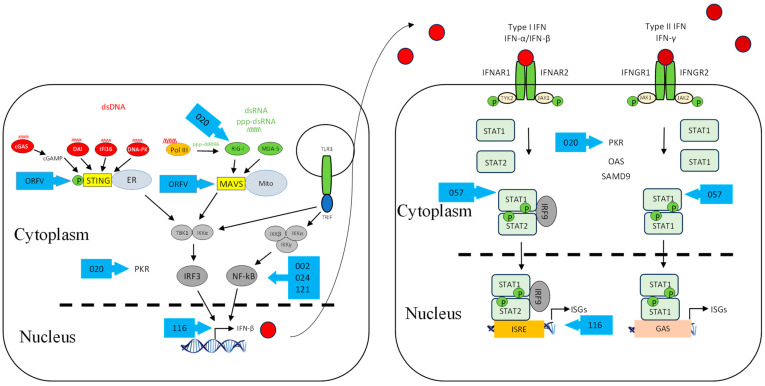
ORFV inhibitors of the interferon induction and signalling pathways. As in Figure 1, shown are DNA- and RNA-sensing mechanisms that lead to IFN signalling. ORFV inhibitors are shown as blue arrows. Further details of the inhibitors are shown in Table 1. Red circle is type I IFN, darker red circle is type II IFN, dashed line is nuclear membrane.

**Figure 3:**
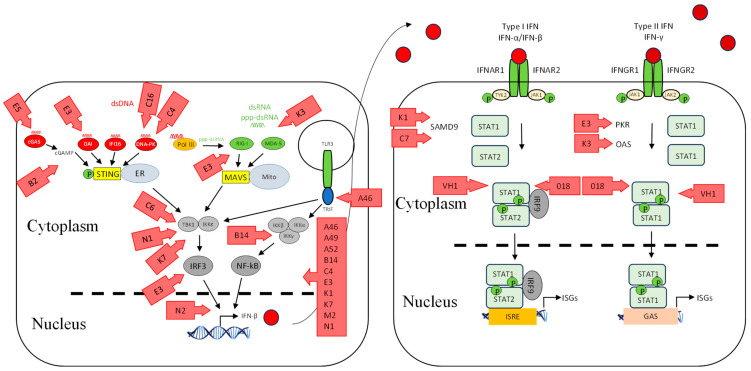
VACV inhibitors of the interferon induction and signalling pathways. As for Figure 1, shown are DNA- and RNA-sensing mechanisms that lead to IFN signalling. VACV inhibitors are shown as red arrows. Further details of the inhibitors are shown in Table 1. Red circle is type I IFN, darker red circle is type II IFN, dashed line is nuclear membrane.

We have recently shown that ORFV employs a strategy that antagonizes the RNA-dependent signalling pathway of IFN induction. The virus potently inhibits the induction of IFN-β upon stimulation with dsRNA via RIG-I-dependent signalling [88]. The exact mechanism underlying this observation is yet to be elucidated, and whether the virus targets upstream at the sensing level or downstream within the signalling pathway. One of the ORFV factors that can be attributed to this dsRNA-dependent signalling inhibition is ORF020. As noted above, it is a homolog of the VACV interferon resistance gene *E3L* that binds dsRNA to prevent the activation of PKR and OAS (Figure 2) [77,78]. Its inhibitory effect on the dsRNA-mediated induction of type I IFN was demonstrated from ectopic vector expression [88,89]. Despite the fact that the exact mechanism of this inhibition is not known, it is likely to be similar to that of VACV E3L [90,91]. VACV E3L sequesters RNA and thus inhibits the dsRNA-mediated induction of type I IFNs (Figure 3) [33,92]. VACV K3L is a PKR inhibitor that has homology to the *n*-terminal region of eIF2α. It is believed that K3L acts as a pseudosubstrate for PKR in lieu of eIF2α (Figure 3) [93,94,95]. Interestingly ORFV does not have a homolog of this gene.

Poxviruses have co-evolved mechanisms to counteract the TLR-dependent response. VACV encodes two proteins known to counteract TLR-mediated signalling, A46R and A52R [96,97], neither of which are encoded by parapoxvirues. They have distinct modes of action and target different cellular proteins involved in TLR signalling (Figure 3). A46R is a TIR domain-containing protein that can associate with several TIR domain-containing adaptors, such as MyD88, TIRAP, TRIF, and TRAM, the cytoplasmic domains of TLRs, leading to the inhibition of TLR-induced NF-κB and IRF3 activation (Figure 3) [98]. A52R has the ability to inhibit TLR-induced NF-κB activation by interacting with IRAK2 and TRAF6 (Figure 3) [99].

Other strategies that poxviruses utilize to avoid RNA sensing is by modifying the structure of their RNA. They can cap the end of their newly synthesized mRNA to mimic cellular RNAs, or even decap them to prevent their accumulation and an antiviral response, as shown in VACV [100,101,102,103].

### 4.2. Evasion of DNA-Dependent Sensing

Primary keratinocytes, in which ORFV replicates, express a number of cytosolic DNA sensors such as ZBP1 (DAI), IFI16, cGAS, STING, and AIM2, in addition to the endosomal TLR9 [15,104,105,106]. Our recent studies have shown that ORFV has evolved a strategy to evade cytosolic DNA-dependent signalling [31]. These results strongly suggest that ORFV encodes factors that interfere with the DNA-dependent signalling pathway of IFN-β expression; however, the underlying mechanism is yet to be determined. Several poxvirus antagonists have been identified that directly interfere with the cytosolic DNA sensing signalling pathways, and VACV in particular devotes a considerable number of factors that target this pathway. VACV E3L contains a Z-DNA-binding domain that can prevent the interaction of DAI with DNA, resulting in the inhibition of DNA-induced IFN-β (Figure 3) [107]. DNA-PK was identified to be targeted by the VACV C16 and C4 proteins in which it binds to the Ku subunit and prevents it from binding to DNA (Figure 3) [108,109]. DNA-PK is a heterotrimeric complex consisting of heterodimer Ku70 and Ku80 and acts as a DNA sensor [37]. Two other factors expressed by VACV were recently discovered to counteract DNA detection. VACV proteins E5 and Poxin counteract cGAS and cGAMP, respectively, by promoting their degradation, which results in STING inhibition (Figure 3) [110,111,112]. From our studies, ORFV appears to target cytosolic DNA sensing similarly to VACV, although homologs of the above VACV genes have not been discovered in ORFV and may suggest that some of the unknown genes in ORFV are involved.

### 4.3. Inhibition of Signalling Molecules

We have recently shown that ORFV, in common with a number of other poxviruses, potently inhibits dsDNA-mediated IFN induction via a STING-dependent pathway, although the underlying mechanism is not known for ORFV (Figure 2) [31]. Georgana, Sumner [113] found that VACV, CPXV, and ECTV, but not MVA, interfere with DNA-induced STING signalling. A number of factors were discovered in VACV that inhibit this pathway. VACV C6 was described as an inhibitor of IFN-β expression by preventing activation of IRF3 and IRF7 at the level of TBK1/IKKε (Figure 3). C6 interacts with subunits of TBK1/IKKε: NAK-associated protein 1 (NAP1), TRAF family member-associated NF-κB activator (TANK), and TBK1 adaptor (SINTBAD), to inhibit IRF3 and IRF7 activation [114]. VACV N1L is another viral protein that has been shown to interfere with DNA sensing by inhibiting the activation of TBK1 (Figure 3). N1L, expressed from MVA, caused a reduction in IFN-β expression, possibly through STING [115]. Furthermore, the induction of IFN-β is inhibited by VACV K7R by binding with DEAD-box protein 3 (DDX3), resulting in the inhibition of TBK1/IKKε-mediated IRF3 activation [116] (Figure 3). VACV B14R interacts with IKKβ and inhibits its phosphorylation, whereas KSHC K13 interacts with the IKKα/IKKβ complex and both result in the inhibition of NF-κB activation (Figure 3) [117,118]. Homologs of the above VACV genes have not been found in ORFV, suggesting that ORFV may have evolved a unique set of genes to inhibit STING-dependent signalling.

The transcription factors that drive the expression of antiviral genes are also key molecules that viruses target. VACV N2 acts downstream of IRF3 phosphorylation and inhibits the activation of IRF3 after translocation into the nucleus through an unknown mechanism (Figure 3) [119]. In addition, VACV E3L has multiple functions including the inhibition of IRF3 and IRF7 activation (Figure 3) [120,121,122].

It is well established that NF-κB drives the expression of proinflammatory cytokines once activated; however, it is also a critical player in regulating the cellular response to IFNs [123]. Accordingly, ORFV and VACV encode a number of proteins that interfere with NF-κB activation. They are early genes and act at different stages in the signalling pathway to inhibit NF-κB activation. ORFV encodes ORF121, ORF002, and ORF024, which inhibit NF-κB-regulated cytokines: IL-1α, IL-6, IL-8, CCL20, CXCL1, CXCL2, CXCL3, ICAM-1, and PTGS2 (Figure 2) [12,124,125]. Bioinformatics analysis has shown that the above ORFV ORFs have no homologs in VACV [9]. VACV encodes A46, A49, A52, B14, C4, E3, K1, K7, M2, and N1 that inhibit NF-κB (Figure 3) [126,127]. It is likely that there are even more NF-κB inhibitors encoded by VACV, as the virus was still able to inhibit NF-κB activation even when all known inhibitors were deleted from the genome [128].

Molluscum contagiosum virus (MCV), a poxvirus that infects humans only and causes a localized skin infection, has developed strategies to evade the innate immune response. MCV expresses multiple proteins that target the signalling pathways, and, interestingly, although MCV is also keratinocyte-tropic, none of these proteins have homologs in ORFV. MC005, MC008, MC132, MC160, and MC159 inhibit NF-κB by targeting different molecules that lead to its activation [129,130,131,132,133]. Moreover, the virus MC159 and MC089 can also target IRF3 and prevent its activation [133,134].

### 4.4. Inhibition of IFN-Induced Signalling

Apart from other biological properties of type I IFNs, i.e., regulating cellular differentiation and proliferation and immunomodulation, they can activate the JAK/STAT signalling pathway [135]. The interaction between IFN-α/β and its receptor (IFNAR1/2) recruits JAK1 and TYK2, leading to activation of the receptor-associated JAK1 and the phosphorylation of STAT 1 and 2. Upon phosphorylation, STAT1 and STAT2 form a heterotrimeric transcription factor complex, IFN-stimulated gene factor 3 (ISGF3) with interferon regulatory factor (IRF-9), which translocates to the nucleus. This trimeric complex binds to the interferon-stimulated response element (ISRE) in the promoters of interferon-stimulated genes (ISGs) to induce their expression (Figure 1). On the other hand, the interaction between IFN-γ and its receptor (IFNGR1/2) recruits JAK1 and JAK2, leading to the activation of STAT1. Upon its phosphorylation, they form a homodimeric complex that translocates to the nucleus. This complex then binds gamma-activated sequence (GAS) of ISGs to drive their expression (Figure 1).

The activity of IFNs is targeted by poxviruses by producing factors that block the IFN-signalling cascade. ORFV modulates the JAK/STAT pathway by dephosphorylating STAT1 at Tyr701 immediately upon infection via a structural protein, ORF057 [136] (Figure 2). ORF057 has 41% amino acid identity with VACV VH1 [8,9,137], also known to dephosphorylate STAT1 (Figure 3) [136,138,139]. In the case of VACV, it was thought that this dephosphorylation activity was due solely to VH1, until the discovery of ORF018 (Figure 3). This factor was shown to bind directly to the SH2 domain of STAT1 and prevent its association with the receptor and, consequently, its phosphorylation [140].

### 4.5. Inhibition of Interferon-Stimulated Genes

As described above, IFNs induce hundreds of antiviral effectors [56,141,142], and poxviruses in particular encode factors that interfere with their actions [127,143].

Recently, a functional analysis of ORF116 encoded by ORFV was investigated in which the transcriptome of HeLa cells infected with either OV-NZ2 wild type or OV-NZ2Δ116 knockout was analyzed by a gene-expression microarray. The microarray data revealed that the expression level of a number of ISGs had been upregulated in the mutant virus-infected cells to higher levels than in wild type-infected cells. These ISGs include IFI44, RIG-I, IFIT2, MDA5, OAS1, OASL, DDX60, ISG20, and IFIT1. The data show that ORF116 has a role in manipulating the antiviral effectors expressed in HeLa cells; however, its direct effect and mechanism are yet to be determined (Figure 2) [144].

Guanylate-binding protein 1 is a large GTPase (GBP1) of the dynamin superfamily and is involved in the regulation of membrane cytoskeleton and cell cycle progression [145]. In many cell types, GBP1 is strongly induced by IFN-γ and acts to reduce cellular proliferation during inflammation [145,146]. Harvey, McCaughan [136] showed that GBP1 is strongly inhibited in ORFV-infected HeLa cells stimulated with IFN-γ. Furthermore, human myxovirus resistance protein A (MxA) is an IFN type l-induced dynamin-like GTPase that protects cells from viral pathogens and is part of the innate immune response and is strongly inhibited in ORFV-infected Hela cells stimulated by IFN-α [136].

VACV KIL and C7L were shown to have a role in inhibiting the IFN effector response by targeting SAMD9 (Figure 3), an ISG that plays a critical antiviral role in viral infection, and the deletion of these two viral genes makes the virus sensitive to IFNs [147,148,149]. ORFV lacks these two genes; however, despite this, ORFV can grow in HeLa cells in which a VACV C7L/K1L deletion mutant does not replicate. Furthermore, ORFV can partially restore the growth of the VACV C7L/K1L deletion mutant in HeLa cells [150], indicating that ORFV encodes factors that are functionally similar to K1L and C7L that subvert the effects of SAMD9.

As described above, a further ORFV factor known to disrupt the activity of ISGs is ORF020, which binds dsRNA to inhibit the activation of PKR [77,78]. More recently, an ORFV factor (ORF129) was discovered that has an inhibitory effect on innate immunity by inhibiting C1QBP, a cellular protein that regulates the immune response [151].

**Table 1:** VACV and ORFV inhibitors.

Signalling Pathway	Protein	References
RNA Sensing	K3	[95,152]
	E3	[33]
	ORF020 *	[77,78]
DNA Sensing	E3	[107]
	C16	[108,109]
	C4	[109]
	E5	[112]
	B2	[110,111]
Signalling Molecules	C6	[114]
	N1	[115,153]
	K7	[116]
	N2	[119]
	E3	[120,121,122]
	ORF002 *	[12,154]
	ORF024 *	[124]
	ORF121 *	[125]
	A46	[97,98]
	A49	[155]
	A52	[97]
	B14	[117,156]
	C4	[157]
	E3	[158]
	K1	[159]
	K7	[160]
	M2	[161]
	N1	[162,163]
IFN-Induced Signalling	ORF057 *	[136]
	VH1	[137,138]
	018	[140]
ISGs	ORF116 *	[144]
	ORF020 *	[77,78]
	K1	[147,148,149]
	C7	[147,148,149]

* indicates protein from ORFV.

## 5. Conclusions and Future Perspectives

Studies in recent years have revealed that ORFV has evolved strategies in common with other poxviruses that counteract immune detection and disrupt the innate response, in particular, the effects of type I IFNs. Although a number of these immune evasion molecules are similar to other poxvirus proteins, many appear to be unique with no homology to other poxvirus genes. We predict that the unexplained inhibitory effects on the viral sensing and modulation of type I IFN signalling pathways observed is due to the unique genes that ORFV encodes. Although the functionality of these genes appears to be conserved, a major shift in their structural evolution could have occurred or the virus could be targeting other factors within signalling pathways. It is also apparent that ORFV encodes far fewer factors than the more virulent poxviruses to subvert the immune response, and this may due to the life cycle of the virus in that it only infects keratinocytes in the infected host and generally causes benign lesions. Further, studies will elucidate the mechanisms that ORFV employs to subvert the host immune response and whether some of the unique genes ORFV encodes have a role.

## Data Availability

No new data were created or analyzed in this study. Data sharing is not applicable to this article.
